# Exploring the genetics of lithium response in bipolar disorders

**DOI:** 10.21203/rs.3.rs-3677630/v1

**Published:** 2023-12-02

**Authors:** Marisol Herrera-Rivero, Mazda Adli, Kazufumi Akiyama, Nirmala Akula, Azmeraw T. Amare, Raffaella Ardau, Bárbara Arias, Jean-Michel Aubry, Lena Backlund, Frank Bellivier, Antonio Benabarre, Susanne Bengesser, Abesh Kumar Bhattacharjee, Joanna M. Biernacka, Armin Birner, Micah Cearns, Pablo Cervantes, Hsi-Chung Chen, Caterina Chillotti, Sven Cichon, Scott R. Clark, Francesc Colom, Cristiana Cruceanu, Piotr M. Czerski, Nina Dalkner, Franziska Degenhardt, Maria Del Zompo, J. Raymond DePaulo, Bruno Etain, Peter Falkai, Ewa Ferensztajn-Rochowiak, Andreas J. Forstner, Josef Frank, Louise Frisén, Mark A. Frye, Janice M. Fullerton, Carla Gallo, Sébastien Gard, Julie S. Garnham, Fernando S. Goes, Maria Grigoroiu-Serbanescu, Paul Grof, Ryota Hashimoto, Roland Hasler, Joanna Hauser, Urs Heilbronner, Stefan Herms, Per Hoffmann, Liping Hou, Yi-Hsiang Hsu, Stéphane Jamain, Esther Jiménez, Jean-Pierre Kahn, Layla Kassem, Tadafumi Kato, John Kelsoe, Sarah Kittel-Schneider, Po-Hsiu Kuo, Ichiro Kusumi, Barbara König, Gonzalo Laje, Mikael Landén, Catharina Lavebratt, Marion Leboyer, Susan G. Leckband, Mario Maj, Mirko Manchia, Cynthia Marie-Claire, Lina Martinsson, Michael J. McCarthy, Susan L. McElroy, Vincent Millischer, Marina Mitjans, Francis M. Mondimore, Palmiero Monteleone, Caroline M. Nievergelt, Tomas Novák, Markus M. Nöthen, Claire O’Donovan, Norio Ozaki, Sergi Papiol, Andrea Pfennig, Claudia Pisanu, James B. Potash, Andreas Reif, Eva Reininghaus, Hélène Richard-Lepouriel, Gloria Roberts, Guy A. Rouleau, Janusz K. Rybakowski, Martin Schalling, Peter R. Schofield, Klaus Oliver Schubert, Eva C. Schulte, Barbara W. Schweizer, Giovanni Severino, Tatyana Shekhtman, Paul D. Shilling, Katzutaka Shimoda, Christian Simhandl, Claire M. Slaney, Alessio Squassina, Thomas Stamm, Pavla Stopkova, Fabian Streit, Fasil Tekola-Ayele, Anbupalam Thalamuthu, Alfonso Tortorella, Gustavo Turecki, Julia Veeh, Eduard Vieta, Biju Viswanath, Stephanie H. Witt, Peter P. Zandi, Martin Alda, Michael Bauer, Francis J. McMahon, Philip B. Mitchell, Marcella Rietschel, Thomas G. Schulze, Bernhard T. Baune

**Affiliations:** University of Münster; Charité - Universitätsmedizin Berlin; Dokkyo Medical University School of Medicine; United States Department of Health and Human Services; University of Adelaide; Hospital University Agency of Cagliari; University of Barcelona, CIBERSAM; Geneva University Hospitals; Karolinska Institutet; Université Paris Cité, Inserm UMR-S 1144; Hospital Clinic, University of Barcelona, IDIBAPS; Medical University of Graz; University of California San Diego; Mayo Clinic; Medical University of Graz; University of Adelaide; McGill University Health Centre; National Taiwan University Hospital; Hospital University Agency of Cagliari; University Hospital of Basel; University of Adelaide; Hospital Del Mar; McGill University; Poznan University of Medical Sciences; Medical University of Graz; University of Bonn; University of Cagliari; Johns Hopkins University; Université Paris Cité, Inserm UMR-S 1144; Ludwig-Maximilian-University Munich; Poznan University of Medical Sciences; University of Bonn; Central Institute of Mental Health, University of Heidelberg; Karolinska Institutet; Mayo Clinic; UNSW Sydney; Cayetano Heredia University; Hôpital Charles Perrens; Dalhousie University; Johns Hopkins University; Alexandru Obregia Clinical Psychiatric Hospital; Mood Disorders Center of Ottawa; National Institute of Mental Health; Geneva University Hospitals; Poznan University of Medical Sciences; Ludwig-Maximilian-University Munich; University of Bonn; University of Bonn; United States Department of Health and Human Services; Harvard University; Paris-Est Créteil University; Hospital Clinic, University of Barcelona, IDIBAPS; Centre Psychothérapique de Nancy - Université de Lorraine; United States Department of Health and Human Services; Juntendo University; University of California San Diego; University Hospital Würzburg; National Taiwan University Hospital; Hokkaido University Graduate School of Medicine; Landesklinikum Neunkirchen; United States Department of Health and Human Services; University of Gothenburg; Karolinska Institutet; Paris-Est Créteil University; VA San Diego Healthcare System; University of Campania ‘Luigi Vanvitelli’; University of Cagliari; Université Paris Cité, Inserm UMR-S 1144; Karolinska Institutet; University of California San Diego; University of Cincinnati; Karolinska Institutet; University of Barcelona; Johns Hopkins University; University of Salerno; University of California San Diego; National Institute of Mental Health; University of Bonn; Dalhousie University; Nagoya University; Ludwig-Maximilian-University Munich; University Hospital Carl Gustav Carus, Technische Universität Dresden; University of Cagliari; Johns Hopkins University; University Hospital Frankfurt; Medical University of Graz; Geneva University Hospitals; UNSW Sydney; McGill University; Poznan University of Medical Sciences; Karolinska Institutet; UNSW Sydney; University of Adelaide; Ludwig-Maximilian-University Munich; Johns Hopkins University; University of Cagliari; University of California San Diego; University of California San Diego; Dokkyo Medical University School of Medicine; Sigmund Freud University Vienna; Dalhousie University; University of Cagliari; Charité - Universitätsmedizin Berlin; National Institute of Mental Health; Central Institute of Mental Health, University of Heidelberg; National Institutes of Health; UNSW Sydney; University of Perugia; McGill University; University Hospital Frankfurt; Hospital Clinic, University of Barcelona, IDIBAPS; National Institute of Mental Health and Neurosciences; Central Institute of Mental Health, University of Heidelberg; Johns Hopkins University; Dalhousie University; University Hospital Carl Gustav Carus, Technische Universität Dresden; United States Department of Health and Human Services; UNSW Sydney; Central Institute of Mental Health, University of Heidelberg; Ludwig-Maximilian-University Munich; University of Münster

**Keywords:** Bipolar disorder, lithium treatment, psychiatric symptoms, comorbidity, genetics

## Abstract

**Background:**

Lithium (Li) remains the treatment of choice for bipolar disorders (BP). Its mood-stabilizing effects help reduce the long-term burden of mania, depression and suicide risk in patients with BP. It also has been shown to have beneficial effects on disease-associated conditions, including sleep and cardiovascular disorders. However, the individual responses to Li treatment vary within and between diagnostic subtypes of BP (e.g. BP-I and BP-II) according to the clinical presentation. Moreover, long-term Li treatment has been linked to adverse side-effects that are a cause of concern and non-adherence, including the risk of developing chronic medical conditions such as thyroid and renal disease. In recent years, studies by the Consortium on Lithium Genetics (ConLiGen) have uncovered a number of genetic factors that contribute to the variability in Li treatment response in patients with BP. Here, we leveraged the ConLiGen cohort (N=2,064) to investigate the genetic basis of Li effects in BP. For this, we studied how Li response and linked genes associate with the psychiatric symptoms and polygenic load for medical comorbidities, placing particular emphasis on identifying differences between BP-I and BP-II.

**Results:**

We found that clinical response to Li treatment, measured with the Alda scale, was associated with a diminished burden of mania, depression, substance and alcohol abuse, psychosis and suicidal ideation in patients with BP-I and, in patients with BP-II, of depression only. Our genetic analyses showed that a stronger clinical response to Li was modestly related to lower polygenic load for diabetes and hypertension in BP-I but not BP-II. Moreover, our results suggested that a number of genes that have been previously linked to Li response variability in BP differentially relate to the psychiatric symptomatology, particularly to the numbers of manic and depressive episodes, and to the polygenic load for comorbid conditions, including diabetes, hypertension and hypothyroidism.

**Conclusions:**

Taken together, our findings suggest that the effects of Li on symptomatology and comorbidity in BP are partially modulated by common genetic factors, with differential effects between BP-I and BP-II.

## BACKGROUND

Lithium (Li) is the first-line maintenance treatment for bipolar disorders (BP). Multiple beneficial properties have been attributed to Li, including mood stabilization, cardio- and neuroprotection, circadian regulation, immunomodulation, and suicide prevention in patients with BP [Geoffroy PA et al., 2016; Volkmann C et al., 2020; Xu N et al., 2021; Queissner R et al., 2021; Miller BJ & McCall WV, 2022; Rybakowski JK, 2022; Chen PH et al., 2023; Szałach ŁP et al., 2023]. Li is not exempt from acute side-effects, the most frequent being gastrointestinal complaints, that may cause non-adherence. However, it is the long-term adverse effects, including thyroid and kidney problems [Volkmann C et al., 2020; Ferensztajn-Rochowiak E et al., 2021], that cause most concern.

Individual responses to Li vary according to the clinical presentation of the disease. Reportedly, only about 30% of patients with BP have a full response to Li treatment. Various clinical, psychosocial and demographic factors that affect Li response have been described [Nunes A et al., 2020; Ferensztajn-Rochowiak E et al., 2021]. Moreover, genetic studies have established Li response as a polygenic trait [Papiol S et al., 2022]. Previous work performed by the Consortium on Lithium Genetics (ConLiGen) has offered significant insights into the molecular mechanisms contributing to Li response [Amare AT et al., 2023], as well as the links with the polygenic scores of other psychiatric disorders [Amare AT et al., 2018; Schubert KO et al., 2021; Coombes BJ et al., 2021] and with suicidal behavior [Yoshida T et al., 2019] in BP. However, the relationships between Li response and disease features, particularly comorbidity, remain largely unexplored. Moreover, most studies have made no distinction between different diagnostic groups. Here, we used data from ConLiGen participants (N = 2,064) to explore how the genetic factors that contribute to Li response variability in patients with BP are associated with specific psychiatric symptoms and the polygenic load (i.e. genetic risk) for medical comorbid conditions, and whether these relationships differ between BP types I and II.

## METHODS

### Study population

The ConLiGen cohort has been described elsewhere [Hou L et al., 2016]. Briefly, between 2003 and 2013, ConLiGen recruited over 2,500 Li-treated individuals with bipolar spectrum disorders at various sites in Europe, the United States, Australia and East-Asia. The inclusion criteria consisted of a diagnosis of bipolar disorder type I (BP-I) or type II (BP-II), schizoaffective bipolar disorder or bipolar disorder not otherwise specified in accordance with the criteria established in the Diagnostic and Statistical Manual of Mental Disorders (DSM) versions III or IV, as well as Li treatment that lasted a minimum of six months with no additional mood stabilizers. Long-term responses to Li treatment were assessed using the Alda scale, where an A subscale rates the degree of response in the range 0–10 and a B subscale reflects the relationship between improvement and treatment. A total score, ranging from 0–10, is obtained by subtracting the B score from the A score [Manchia M et al., 2013]. Negative scores are set to 0. Here, we used a sample of 2,064 ConLiGen participants with complete covariate phenotypes: sex, age-at-onset (AAO), age at recruitment (i.e. sample collection), diagnosis and recruitment site (used to establish population).

The Ethics Committee at the University of Heidelberg provided central approval for ConLiGen. Written informed consent from all participants was obtained according to the study protocols of each of the participating sites and their institutions. All procedures were performed in accordance with the guidelines of the Declaration of Helsinki.

### Genotype data

Genotyping, quality control (QC) and imputation of the ConLiGen cohort has been described elsewhere [Hou L et al., 2016]. Briefly, DNA genotyping by array was performed from peripheral blood samples in two batches of similar composition, originally referred to as “GWAS1” (N = 1,162) and “GWAS2” (N = 1,401). Standard procedures for QC and imputation using the 1000 Genomes Project reference panel were employed. Here, we used an updated ConLiGen dataset we previously described in detail [Herrera-Rivero M et al., 2023], in which we re-imputed the combined ConLiGen batches using the Haplotype Reference Consortium (HRC) panel. This procedure increased the number of markers and the quality of the dataset, increasing its suitability for polygenic score (PGS) analyses. Single nucleotide polymorphisms (SNPs) in 37 genes that were previously reported to contribute to Li response in ConLiGen following a gene-level genome-wide analysis [Amare et al., 2023] were extracted from the dataset using a window of ± 1 kb from the start and end positions of the gene (according to the Ensembl hg19 genome build). Our final dataset contained 9,374 SNPs corresponding to 34 Li response-linked genes and 2,064 individuals with BP, from which 1,669 had a diagnosis of BP-I and 370 of BP-II.

### Phenotypes

Li response: We used the total Alda score as a measure of Li response. This was available for all 2,064 individuals included in our study.

Psychiatric symptoms: Here, the psychiatric symptoms corresponded to the numbers of episodes of depression and mania, the presence of psychosis, alcohol and substance abuse, and of suicidal ideation. These variables were available for a maximum of 853 individuals from the GWAS1 batch.

Genetic risk for medical comorbidities: Based on the literature, we identified various conditions that are comorbid in BP and searched the PGS Catalog [Lambert SA et al., 2021] for publicly available PGSs for these. Weight files for the calculation of PGSs for various traits, such as disorders of sleep and metabolism, were downloaded from the PGS Catalog and used for allelic scoring in the total ConLiGen sample with plink 1.9 [Chang CC et al., 2015]. Standardized sum scores were used for analysis. Because of incomplete compatibility between PGS SNPs and variants in the ConLiGen dataset, only PGSs with compatibility > 78% were used. These corresponded to the following traits: chronotype (PGS ID: PGS002209), sleep duration (PGS ID: PGS002196), insomnia (PGS ID: PGS002149), hypertension (PGS ID: PGS002047), hypothyroidism (PGS ID: PGS001816) and type 2 diabetes (PGS ID: PGS003118) [Privé F et al., 2022; Ma Y et al., 2022] (Suppl.Table.1). Traits excluded due to lower compatibility included cardiovascular disorders, obesity, migraine and asthma.

### Statistical analyses

Associations between total Alda scores and psychiatric symptoms were tested using robust linear/logistic regression models with the “robustbase” R package (n_max_=853). Models were adjusted for sex, AAO and age. Associations between total Alda scores and PGSs for comorbid conditions were tested using partial Spearman correlation with the “ppcor” R package (n_max_=2,064). Models were adjusted for sex, AAO, age and population. SNP-phenotype associations were tested using linear/logistic regression models with plink 1.9. Models were adjusted for sex, AAO, age, population, total Alda score and the first eight dimensions coming from a principal components analysis of the genotypes. When testing associations using all individuals, all models were also adjusted for the differential BP diagnosis. All associations were also tested separately for BP-I and BP-II. For exploratory purposes, significance was set to nominal (i.e. unadjusted) p < 0.05 and p < 0.01 for total Alda score and SNP-phenotype associations, respectively.

## RESULTS

To explore how Li response genes are associated with specific psychiatric symptoms and the poygenic load for medical comorbid conditions, and whether these relationships differ between BP types I and II, we used a sample of 2,064 individuals with BP from the ConLiGen cohort. From these, 1,197 (58%) were females, 1,669 (80.1%) had a diagnosis of BP-I and 370 (17.9%) were diagnosed with BP-II. The mean AAO in the sample was 25 ± 11 years, while the mean age at recruitment was 47 ± 14 years. The mean total Alda score was 4.22 ± 3.16 points, with 29.8% of the patients being categorized as good responders (total Alda score ≥ 7). Compared to BP-I, BP-II patients were slightly older at disease onset (28 ± 12 vs 24 ± 10 years) and recruitment (50 ± 14 vs 47 ± 14 years), and had higher rates of females (61.9% vs 57.2%) and good Li responders (34.1% vs 28.2%). However, the mean total Alda scores were very similar (4.6 ± 3.2 vs 4.2 ± 3.1 points).

**Figure 1:**
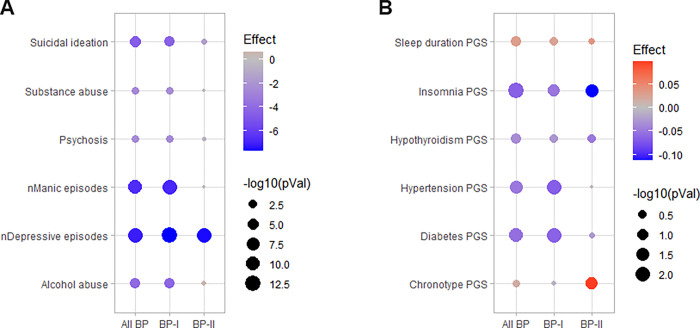
Links between phenotypes and Li responses in ConLiGen. **A)** Association test results between total Alda scores and psychiatric symptoms. Shown are the nominal p-values (−log10) and z-values (effect) obtained from robust linear/logistic regression models. **B)** Correlation test results between total Alda scores and PGSs for comorbid conditions. Shown are the nominal p-values (−log10) and correlation coefficients (effect) obtained from partial correlation models using the Spearman method.

First, we explored the association between Li response and psychiatric symptoms/PGSs for comorbid conditions. Using a nominal significance threshold (p < 0.05), we found that the total Alda scores showed a negative relationship with all psychiatric symptom variables in all BP (n_max_=835) and BP-I (n_max_=665) individuals. However, in BP-II individuals (n_max_=153), the total Alda scores showed a negative relationship only with the number of depressive episodes (Fig. 1A). These observations suggest that better responses to Li treatment diminish the burden of most psychiatric symptoms in patients with BP-I, but only that of depression in patients with BP-II. Noticeably, these results survived false discovery rate correction (FDR < 0.05). Furthermore, the total Alda scores also correlated negatively with the PGSs for diabetes and hypertension in all BP (N = 2,064) and BP-I (N = 1,669) individuals, and with the PGS for insomnia in all BP, BP-I and BP-II (N = 370) individuals (Fig. 1B). This suggested that better Li response correlates with lower genetic burden predisposing to insomnia in patients with BP in general, and to diabetes and hypertension in patients with BP-I diagnosis in particular. However, none of the nominal associations with PGSs survived FDR correction in our sample.

**Figure 2:**
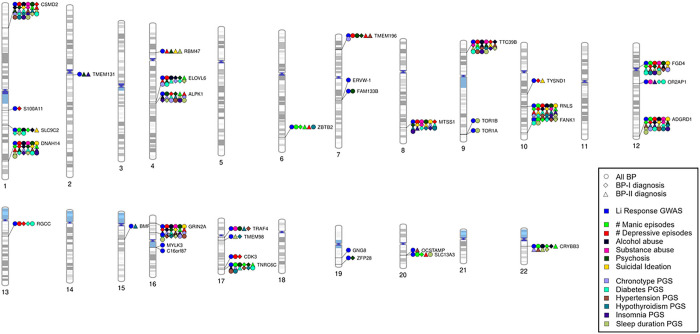
Visual integration of nominal findings for Li response genes. Shapes depict the diagnostic group analyzed while colors refer to the phenotypes nominally associated with the gene in our analyses, except for the blue color, which localized even the genes not analyzed in this study that were reported by Amare et al., 2023 as contributors to Li response in ConLiGen.

Second, we explored the association between genes previously linked to Li response and psychiatric symptoms/PGSs for comorbid conditions. Using a nominal significance threshold (p < 0.01) as indicative of suggestive association, we found that 32 of the 34 genes tested were suggested to associate with specific psychiatric symptoms and/or PGSs for comorbid conditions (Fig. 2, Suppl.Tables.2–7). The most significant hits were for the number of manic episodes, with *SLC13A3* as top gene in BP-I and *TNRC6C* in BP-II, followed by the number of depressive episodes, with *MTSS1* as top gene in BP-I and *DNAH14* in BP-II (Table 1). These results suggest some candidate genes that might be involved in Li effects with respect to episodes of mania and depression in BP.

Taken together, 22 of the 34 genes tested were nominally associated with at least one psychiatric symptom and one PGS in at least one of the tests performed (i.e. all BP, BP-I and BP-II). Noticeably, some of the Li response genes were suggested to associate with all the phenotypes that we studied in at least one of the tests. We also observed that genes with the most overlaps, including *RNLS, GRIN2A, CSMD2, DNAH14* and *TTC39B* (Table 2), represented the most significant hits obtained in BP-I or BP-II for various PGSs for comorbid conditions (Table 1). This suggests that Li effects on medical comorbid conditions might also involve shared genetic factors, although with small independent effects.

Overall, our results suggested that genes linked to Li response also contribute to modulate the clinical presentation of BP, and that these contributions vary between BP-I and BP-II diagnoses in many instances. We corroborated the latter observation by looking into the overlapping and non-overlapping genes between the BP-I and BP-II analyses (Table 3). Here, we observed that, for example, *GRIN2A* was suggested to relate to the number of depressive episodes, the presence of alcohol abuse, and the polygenic contribution to chronotype, diabetes and hypertension in both major types of BP. However, it was suggested to be linked to the presence of psychosis and suicidal ideation, and the polygenic contribution to sleep duration and hypothyroidism in BP-I only, while relating to the number of manic episodes and the genetic load for insomnia only in BP-II.

## DISCUSSION

We showed that positive responses to Li treatment in patients with BP are generally more beneficial to those patients diagnosed with BP-I than to those with a BP-II diagnosis, and that genes linked to Li response also contribute to the clinical presentation of the disorder in terms of psychiatric symptomatology and, potentially, the risk of medical comorbid conditions. This may partly explain why Li responses usually vary according to clinical features, and why clinical and psychosocial factors can only partially predict Li responses [Tondo L et al., 2001; Ferensztajn-Rochowiak E et al., 2021].

Often, the efficacy of Li treatment in BP is assessed without making distinction between BP types and/or is focused on manic-depressive episodes, with disregard of other disease-associated afflictions. However, it is plausible that the beneficial effects of Li treatment on psychiatric symptomatology are related to its effects on other health issues associated with BP, such as improving inflammation and sleep [Geoffroy PA et al., 2016; Szałach ŁP et al., 2023]. Moreover, some studies have shown that Li impacts differently the frequency and duration of mood episodes in BP-I and BP-II [Tondo L et al., 2001], which might relate to stronger effects on acute manic than depressive episodes [Fountoulakis KN et al., 2022]. The results of our genetic study are in agreement with such observations. Indeed, we found that Li response genes were more strongly associated with manic than depressive episodes in both BP-I and BP-II. In addition, Li response genes were modestly but differentially associated with other features relevant to the clinical presentation, including, for example, suicidal ideation, psychosis and polygenic load for insomnia and hypothyroidism, in both BP-I and BP-II. Nevertheless, the fact that the results of our genetic analyses did not exactly match those obtained for the total Alda score, where the positive effects of Li showed a clear bias towards BP-I, also suggest important gene-environment interactions.

Despite the exploratory character of our genetic study, we believe that it also offers some valuable insights into the molecular mechanisms underlying inter-individual variability in Li response. For example, renalase (*RNLS*) was one of the most highlighted genes in our study. In addition to its link to Li response in BP [Amare AT et al., 2023], serum renalase levels have been reported to be lower in patients with schizophrenia (SCZ) than in control individuals [Catak Z et al., 2019], and Li response was previously shown to inversely associate with the genetic risk for SCZ [Amare AT et al., 2018]. *RNLS* is thought to modulate blood pressure and cardiac function, and has been associated with metabolic and cardiovascular alterations as well as kidney disease [Vijayakumar A & Mahapatra NR, 2022], all of which are affected by Li. Similar are the cases of *CSMD2* and *GRIN2A*, which are involved in the control of the complement cascade and N-methyl-D-aspartate (NMDA) receptor activity, respectively. Polymorphisms in both genes have also been associated with SCZ [Tang J et al., 2006; Håvik B et al., 2011] and their respective functions are reported targets of Li effects [Ghasemi M & Dehpour AR, 2011; Yu Z et al., 2015].

## CONCLUSIONS

Taken together, our findings suggest that the effects of Li on symptomatology and comorbidity in BP are partially modulated by common genetic factors, with differential effects between BP-I and BP-II. These findings might pave the way towards the development of more personalized treatment strategies for patients with BP.

## Figures and Tables

**Table 1:** Phenotype-based summary of findings for the association analyses between Li response genes and psychiatric symptoms/PGSs for comorbid conditions in ConLiGen.

Phenotype	N	# Cases	# Controls	# SNPs p <0.01	# Genes	Top gene	Top# SNPs p< 0.01	Lowest p
*All BP*								
# Manic episodes	724	-	-	38	9	*SLC13A3*	11	2.48E-08
# Depressive episodes	789	-	-	225	12	*FGD4*	75	5.15E-06
Alcohol abuse	835	140	695	114	9	*ELOVL6*	5	1.11E-04
Substance abuse	832	135	697	143	9	*ADGRD1*	45	4.17E-04
Psychosis	692	342	350	55	11	*GRIN2A*	12	7.83E-04
Suicidal ideation	660	321	339	10	6	*DNAH14*	1	2.31E-03
Insomnia PGS	2064	-	-	57	8	*CSMD2*	6	1.73E-04
Sleep duration PGS	2064	-	-	211	12	*DNAH14*	133	1.12E-04
Chronotype PGS	2064	-	-	81	7	*GRIN2A*	47	4.06E-04
Diabetes PGS	2064	-	-	111	12	*CSMD2*	33	6.28E-04
Hypertension PGS	2064	-	-	34	7	*TTC39B*	5	9.57E-05
Hypothyroidism PGS	2064	-	-	82	7	*MTSS1*	42	4.73E-04
*BP-I diagnosis*								
# Manic episodes	641	-	-	48	10	*SLC13A3*	11	2.15E-08
# Depressive episodes	632	-	-	193	13	*MTSS1*	12	1.52E-06
Alcohol abuse	665	129	536	131	9	*CSMD2*	52	1.34E-04
Substance abuse	662	121	541	121	5	*ADGRD1*	52	4.13E-04
Psychosis	564	318	246	87	10	*CSMD2*	21	7.17E-04
Suicidal ideation	530	264	266	41	6	*MTSS1*	1	2.15E-04
Insomnia PGS	1669	-	-	48	6	*ALPK1*	4	3.92E-04
Sleep duration PGS	1669	-	-	174	11	*RNLS*	3	4.37E-05
Chronotype PGS	1669	-	-	35	5	*RNLS*	2	1.76E-04
Diabetes PGS	1669	-	-	74	13	*TTC39B*	1	6.78E-04
Hypertension PGS	1669	-	-	29	7	*TTC39B*	1	6.81E-04
Hypothyroidism PGS	1669	-	-	38	8	*CSMD2*	4	6.95E-04
*BP-II diagnosis*								
# Manic episodes	68	-	-	113	10	*TNRC6C*	3	3.76E-79
# Depressive episodes	141	-	-	128	11	*DNAH14*	6	3.12E-08
Alcohol abuse	153	7	146	7	5	*TNRC6C*	2	1.80E-03
Substance abuse	153	8	145	0	0	-	-	-
Psychosis	115	12	103	353	7	*TMEM131*	46	1.08E-03
Suicidal ideation	118	48	70	79	7	*TTC39B*	24	2.49E-03
Insomnia PGS	370	-	-	209	7	*GRIN2A*	38	2.65E-04
Sleep duration PGS	370	-	-	64	9	*DNAH14*	16	2.95E-04
Chronotype PGS	370	-	-	32	7	*GRIN2A*	19	1.81E-03
Diabetes PGS	370	-	-	97	9	*MTSS1*	6	2.01E-04
Hypertension PGS	370	-	-	130	10	*TMEM196*	27	3.21E-04
Hypothyroidism PGS	370	-	-	70	7	*BMF*	12	1.92E-04

**Table 2:** Gene-based summary of findings for the association analyses between Li response genes and psychiatric symptoms/PGSs for comorbid conditions in ConLiGen.

Gene	Chr	Gene start (−1kb)	Gene end (+ 1kb)	# tested SNPs	Psychiatric phenotype count			PGS phenotype count			Max. # phenotypes
					All	BP-I	BP-II	All	BP-I	BP-II	
*CSMD2*	1	33978609	34632443	1064	4	5	3	5	5	5	12
*S100A11*	1	152003982	152021383	14	0	1	0	0	0	0	1
*SLC9C2*	1	173468603	173573233	179	2	2	1	1	2	0	5
*DNAH14*	1	225082964	225587996	1417	5	5	3	3	3	5	11
*TMEM131*	2	98371799	98613388	358	0	0	1	0	0	1	2
*RBM47*	4	40424272	40633892	164	0	0	3	0	0	1	4
*ELOVL6*	4	110966002	111121355	261	2	2	3	2	3	1	7
*ALPK1*	4	113205665	113364776	301	1	2	3	4	3	4	10
*ZBTB2*	6	151684252	151713683	43	1	1	2	1	0	0	3
*TMEM196*	7	19757933	19814221	108	2	1	1	1	0	1	4
*ERVW-1*	7	92096694	92108300	19	0	0	0	0	0	0	0
*FAM133B*	7	92189107	92220708	50	1	0	0	0	0	0	1
*MTSS1*	8	125562031	125741730	499	4	4	1	2	3	4	8
*TTC39B*	9	15162620	15308358	408	3	3	4	4	5	2	11
*TOR1B*	9	132564432	132574560	20	0	0	0	1	0	0	1
*TOR1A*	9	132574223	132587413	32	0	0	0	1	0	0	1
*TYSND1*	10	71896737	71907432	40	0	1	1	0	0	0	2
*RNLS*	10	90032621	90345287	628	5	5	3	6	6	4	12
*FANK1*	10	127584108	127699161	250	1	1	0	2	3	0	4
*FGD4*	12	32551463	32799984	882	5	3	3	5	2	3	12
*OR2AP1*	12	55967199	55970128	7	1	0	0	1	1	1	3
*ADGRD1*	12	131437452	131627014	603	5	5	1	6	3	4	12
*RGCC*	13	42030695	42046018	35	1	1	0	1	1	0	2
*BMF*	15	40379091	40402093	16	0	0	0	0	0	1	1
*GRIN2A*	16	9851376	10277611	1624	5	4	3	3	5	4	12
*CHP2*	16	23764948	23771272	10	0	0	0	0	0	0	0
*MYLK3*	16	46739891	46825319	0	0	0	0	0	0	0	0
*C16orf87*	16	46829519	46866323	0	0	0	0	0	0	0	0
*TRAF4*	17	27070002	27078974	8	2	0	0	0	1	1	4
*TMEM98*	17	31253928	31273124	33	0	0	0	0	1	1	2
*CDK3*	17	73995987	74003080	4	1	1	0	0	0	0	1
*TNRC6C*	17	75999249	76105916	153	2	2	2	3	2	2	7
*GNG8*	19	47136333	47138942	0	0	0	0	0	0	0	0
*ZFP28*	19	57049317	57069169	46	0	1	0	0	0	0	1
*OCSTAMP*	20	45168585	45180213	10	0	0	0	0	0	1	1
*SLC13A3*	20	45185463	45305714	58	1	1	1	1	0	0	3
*CRYBB3*	22	25594817	25604330	31	2	2	1	0	1	3	5

**Table 3:** Li response genes nominally associated with psychiatric symptoms/PGSs for comorbid conditions in ConLiGen. Shown are the overlapping and non-overlapping genes between BP-I and BP-II diagnostic groups.

Phenotype	BP-I only	BP-II only	Overlap
# Manic episodes	*ADGRD1, FANK1, FGD4, SLC13A3, SLC9C2*	*ALPK1, CSMD2, ELOVL6, GRIN2A, TTC39B*	*CRYBB3, DNAH14, RNLS, TNRC6C, ZBTB2*
# Depressive episodes	*ADGRD1, CDK3, MTSS1, RGCC, S100A11, TTC39B, TYSND1*	*ELOVL6, RBM47, SLC13A3, TMEM196, ZBTB2*	*ALPK1, CSMD2, DNAH14, FGD4, GRIN2A, RNLS*
Alcohol abuse	*ADGRD1, CRYBB3, CSMD2, DNAH14, ELOVL6, RNLS, SLC9C2*	*ALPK1, FGD4, TNRC6C*	*GRIN2A, TTC39B*
Substance abuse	*ADGRD1, CSMD2, MTSS1, RNLS, TTC39B*	-	-
Psychosis	*ALPK1, FGD4, GRIN2A, MTSS1, TMEM196, TNRC6C, ZFP28*	*ADGRD1, RBM47, TMEM131, TTC39B*	*CSMD2, DNAH14, ELOVL6*
Suicidal ideation	*ADGRD1, CSMD2, DNAH14, GRIN2A, MTSS1*	*FGD4, MTSS1, RBM47, SLC9C2, TTC39B, TYSND1*	*RNLS*
Insomnia PGS	*ALPK1, CSMD2, TTC39B*	*ADGRD1, GRIN2A, OR2AP1, TMEM131*	*DNAH14, MTSS1, RNLS*
Sleep duration PGS	*ELOVL6, FANK1, GRIN2A, RNLS, SLC9C2*	*FGD4, RBM47, TMEM98*	*ADGRD1, ALPK1, CRYBB3, CSMD2, DNAH14, TTC39B*
Chronotype PGS	*ELOVL6, RNLS*	*CRYBB3, DNAH14, MTSS1, TNRC6C*	*ALPK1, CSMD2, GRIN2A*
Diabetes PGS	*ADGRD1, FANK1, OR2AP1, SLC9C2, TTC39B*	*ALPK1, TNRC6C*	*CSMD2, DNAH14, ELOVL6, FGD4, GRIN2A, MTSS1, RNLS*
Hypertension PGS	*FANK1, TNRC6C, TRAF4*	*ADGRD1, CRYBB3, CSMD2, DNAH14, OCSTAMP, TMEM196*	*FGD4, GRIN2A, RNLS, TTC39B*
Hypothyroidism PGS	*GRIN2A, TMEM98, TNRC6C, TTC39B*	*ALPK1, BMF, TRAF4*	*ADGRD1, CSMD2, MTSS1, RNLS*

## Data Availability

The data that support the findings of this study are available from ConLiGen, but restrictions apply to their availability.

## Abbreviations

AAO: age at disease onset
BP: bipolar disorders
ConLiGen: Consortium on Lithium Genetics
Li: lithium
PGS: polygenic score
SNP: single nucleotide polymorphism
